# William Raeburn Jenkins

**Published:** 1890-09

**Authors:** 


### NECROLOGY.

### WILLIAM RAEBURN JENKINS.

It is with extreme regret that we note the death of the young and enterprising publisher and bookseller, William R. Jenkins, on June 16th. He was born in New York city in 1848, and after passing through the common schools he obtained a position in the office of the New York *Herald*. Working his way up into the editorial staff, he in time became the dramatic critic of the *Evening Telegram*, which position he held for nine years. About twelve years ago he opened a modest retail bookstore on Sixth Avenue. By hard work and a close study of the trade he succeeded in developing his store until in time it became not only the largest bookstore but the largest business of any kind in the neighborhood where he had settled. Recognizing the advantage of controlling a specialty, he began in 1884 the publication of veterinary text-books, in which line he was quite successful. Shortly after he began the publication of French and Italian text-books, which had a ready sale from the start. The book trade sustains a real loss in the death of Mr. Jenkins. He was a man in love with the profession, who in years to come would have become one of its mainstays. Young as he was, his enterprise, fair dealing and unselfish devotion to his calling carried his name across the country, and made it famous for qualities which, we regret to say, are becoming rarer from decade to decade.

